# Molecular Surveillance of True Nontypeable *Haemophilus influenzae*: An Evaluation of PCR Screening Assays

**DOI:** 10.1371/journal.pone.0034083

**Published:** 2012-03-28

**Authors:** Michael J. Binks, Beth Temple, Lea-Ann Kirkham, Selma P. Wiertsema, Eileen M. Dunne, Peter C. Richmond, Robyn L. Marsh, Amanda J. Leach, Heidi C. Smith-Vaughan

**Affiliations:** 1 Menzies School of Health Research, Charles Darwin University, Darwin, Australia; 2 School of Paediatrics and Child Health, University of Western Australia, Perth, Australia; 3 Telethon Institute for Child Health Research, Centre for Child Health Research, University of Western Australia, Perth, Australia; 4 Murdoch Childrens Research Institute, Melbourne, Australia; Monash University, Australia

## Abstract

**Background:**

Unambiguous identification of nontypeable *Haemophilus influenzae* (NTHi) is not possible by conventional microbiology. Molecular characterisation of phenotypically defined NTHi isolates suggests that up to 40% are *Haemophilus haemolyticus* (Hh); however, the genetic similarity of NTHi and Hh limits the power of simple molecular techniques such as PCR for species discrimination.

**Methodology/Principal Findings:**

Here we assess the ability of previously published and novel PCR-based assays to identify true NTHi. Sixty phenotypic NTHi isolates, classified by a dual 16S rRNA gene PCR algorithm as NTHi (n = 22), Hh (n = 27) or equivocal (n = 11), were further characterised by sequencing of the 16S rRNA and *recA* genes then interrogated by PCR-based assays targeting the *omp P2*, *omp P6*, *lgt*C, *hpd*, 16S rRNA, *fucK* and *iga* genes. The sequencing data and PCR results were used to define NTHi for this study. Two *hpd* real time PCR assays (*hpd#1* and *hpd#3*) and the conventional *iga* PCR assay were equally efficient at differentiating study-defined NTHi from Hh, each with a receiver operator characteristic curve area of 0.90 [0.83; 0.98]. The *hpd#1* and *hpd#3* assays were completely specific against a panel of common respiratory bacteria, unlike the *iga* PCR, and the *hpd#3* assay was able to detect below 10 copies per reaction.

**Conclusions/Significance:**

Our data suggest an evolutionary continuum between NTHi and Hh and therefore no single gene target could completely differentiate NTHi from Hh. The *hpd#3* real time PCR assay proved to be the superior method for discrimination of NTHi from closely related *Haemophilus* species with the added potential for quantification of *H. influenzae* directly from specimens. We suggest the *hpd#3* assay would be suitable for routine NTHi surveillance and to assess the impact of antibiotics and vaccines, on *H. influenzae* carriage rates, carriage density, and disease.

## Introduction

Globally, respiratory diseases cause an estimated 1.9 million deaths per year [1]. One of the most important aetiological organisms of both adult and childhood respiratory disease is non-typeable *Haemophilus influenzae* (NTHi) [2]. NTHi is frequently isolated from the respiratory tract during episodes of sinusitis, otitis media and pneumonia and is the most common cause of chronic obstructive pulmonary disease and bronchiectasis exacerbations [2], [3], [4].

Traditionally, *Haemophilus* species have been differentiated by growth requirements, including X (Hemin) and V (Nicotinamide) factors, and phenotypic traits such as hydrogen sulphide production, ornithine decarboxylase production and haemolytic activity [5]. Serological methods such as latex and slide agglutination, or PCR assays targeting genes involved with capsule production such as the *bex* genes [6], are used to identify encapsulated strains of *H. influenzae*. Unfortunately, conventional microbiology does not readily distinguish NTHi from its close relative *Haemophilus haemolyticus* (Hh). Haemolysis of horse or rabbit blood agar plates provides the simplest phenotypic difference, although the use of these blood plates for NTHi identification is not commonplace. Furthermore, with the discovery of the non-haemolytic *Haemophilus haemolyticus* phenotype [5], accurate identification of NTHi has become increasingly difficult.

Outside whole genome sequencing, multilocus sequence analysis provides the most accurate identification of true NTHi [7], [8]. However, this technique is both expensive and labour intensive and is not practical for routine screening. The identification of a single gene target for simple consistent identification of NTHi from Hh and variant species would be useful for surveillance and intervention studies to determine the true burden of disease caused by NTHi; however, absolute discrimination of these species may not be possible with this simple strategy.

Several gene targets have been explored for speciation of NTHi, Hh and closely related variants, with varying results. These include the lipo-oligosaccharide gene *lgtC* [7], the IgA protease gene *iga* [7], [8], the fuculose kinase gene *fucK* [9], the pilus gene *pilA* [10] and the 16S rRNA gene [11]. Recently, Wang *et al.* exploited the species heterogeneity of the protein D gene (*hpd*) to develop two probe-based real time PCR assays (*hpd#1* and *hpd#3*). These *hpd#1* and *hpd#3* assays demonstrated excellent sensitivity against a clinically diverse collection of 102 NTHi isolates (96% and 98% respectively) [12]. Two outer membrane protein genes of NTHi (*omp P6* and *omp P2*), are well conserved and have also been used as PCR targets. However, a real time PCR assay targeting the *omp P6* gene was unable to differentiate NTHi from Hh [13], and a recent study demonstrated limited NTHi sensitivity of an *omp P2* real time PCR [12].

Molecular analysis of NTHi collections has revealed a significant presence of Hh in some studies. Among phenotypic NTHi isolates obtained from adult chronic obstructive pulmonary disease patients in the United States, 27% (n = 44) from the nasopharynx and 40% (n = 258) from sputum were found to be Hh using 16S rRNA gene PCR [11]. The same method was used to scrutinise 266 phenotypically-defined NTHi isolates obtained from the nasopharynx of otitis prone and control children (aged 6 to 36 months) in Western Australia. In this study, 79% were designated true NTHi, 12% were Hh and 9% were indeterminate [14]. In contrast, a Danish study re-examined 480 predominantly non-invasive but clinically-associated *H. influenzae* isolates identified by dominant microbiological growth, using probe hybridisation with *fucK*, the adherence and penetration protein gene *hap* and the superoxide dismutase gene *sodC* gene, and had only to reclassify 0.4% (2/480) as Hh [9]. Of the 250 middle ear isolates cultured in the aforementioned studies, none were demonstrated to be Hh or variant strains [9], [11], [14].

In remote Indigenous communities of the Northern Territory, otitis media affects approximately 90% of children less than 2 years of age [15], and 20% of children are hospitalised with an acute lower respiratory infection in their first year of life [16]. In this population NTHi is a more dominant pathogen in terms of prevalence than either *Streptococcus pneumoniae* or *Moraxella catarrhalis* [17], [18] with nasopharyngeal isolation in up to 80% of Indigenous children 3–7 years of age [19], and ear discharge isolation in 21% of Indigenous children with chronic suppurative otitis media (mean age 8 years) [20]. Where there is a high burden of respiratory disease, it is of particular interest to be able to determine the relative contribution of true NTHi and Hh.

Although numerous potential gene targets have been evaluated, definitive identification of NTHi from Hh and variant species with a single gene target has not been demonstrated. Furthermore, there has been no direct comparison of many of the targets investigated to date. In this paper, we assess and compare the ability of a selection of existing and novel PCR-based assays to identify true NTHi from a genetically diverse selection of phenotypic NTHi isolates.

## Results

Sixty phenotypic NTHi isolates, classified by 16S rRNA gene PCR [11] as NTHi (n = 22), Hh (n = 27) or equivocal (n = 11), were further characterised by sequencing of the 16S rRNA and recombinase A (*recA*) genes then interrogated with a selection of PCR-based assays designed to exclusively identify NTHi.

### Sequence phylogeny

**Figure 1:**
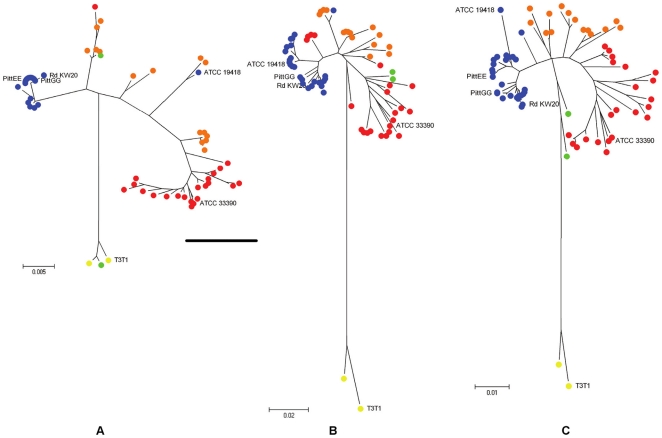
Sequencing phylogeny of study NTHi isolates. Radiation trees are presented for (A) 598 bases of the 16S rRNA gene, (B) 545 bases of the *recA* gene, and (C) the concatenation of these sequences (1143 bases). The trees are rooted by *H. parainfluenzae* T3T1 as indicated by yellow dots. Blue dots represent isolates that cluster with *H. influenzae* reference strains, orange dots represent closely related phylogenetic variants, red dots represent likely Hh isolates and green dots represent variants related to *H. parainfluenzae*. Colours were assigned based on the phylogenetic grouping of the concatenated sequences in radial tree C.

Sequencing of the 16S rRNA and *recA* genes was performed on the 60 study isolates and 2 reference isolates (*H. influenzae* - ATCC 19418, and *H. haemolyticus* - ATCC 33390) respectively yielding 598 and 543 unambiguous bases. Accession numbers are provided in Table S1. Using the Neighbour-Joining algorithm, radial phylogenetic trees (Figure 1) were constructed from the individual and concatenated 16S rRNA and *recA* gene sequences. Six Genbank sequences (Table 1) were included for reference and all trees were rooted by the Genbank reference sequence of *Haemophilus parainfluenzae* – T3T1.

**Table 1:** Genbank reference strains.

			Genbank nucleotide position	
Reference Sequence	Serotype	Accession	16S (598 bp)	*recA* (543 bp)
*H. influenzae* 86-028NP	NTHi	CP000057	193310–193907	685751–686294
*H. influenzae* Rd KW20	d	L42023	127271–127868	621954–622498
*H. influenzae* 10810	b	FQ312006	179410–180007	731026–730483
*H. influenzae* PittGG	NTHi	CP000672	443167–443764	1141114–1141657
*H. influenzae* PittEE	NTHi	CP000671	377847–377250	1805263–1805807
*H. parainfluenzae* T3T1	na	FQ31200	2078840–2079437	662626–663168

Sequence information was obtained from 6 reference strains (www.ncbi.nlm.nih.gov – December 2010) to facilitate phylogenetic speciation. na = not applicable.

For each tree, distally discrete clusters of NTHi and Hh were evident, interspersed by less well defined isolates as shown in Figure 1. The sequenced *H. influenzae* reference strain (ATCC 19418) grouped with the strict cluster of NTHi's by *recA* sequence but was less well defined by the 16S rRNA gene sequence; however, its sequence similarity across both 16S rRNA and *recA* gene sequences was sufficient to place it with the strict NTHi's on the concatenated tree. Also on the concatenated tree two study isolates (40 and 41) diverged toward the *H. parainfluenzae* (T3T1) root isolate, and one isolate (38) grouped with the T3T1 root isolate (Figure 1). The diversity revealed in the phylogeny precluded complete dichotomous speciation.

### PCR of study isolates

**Figure 2:**
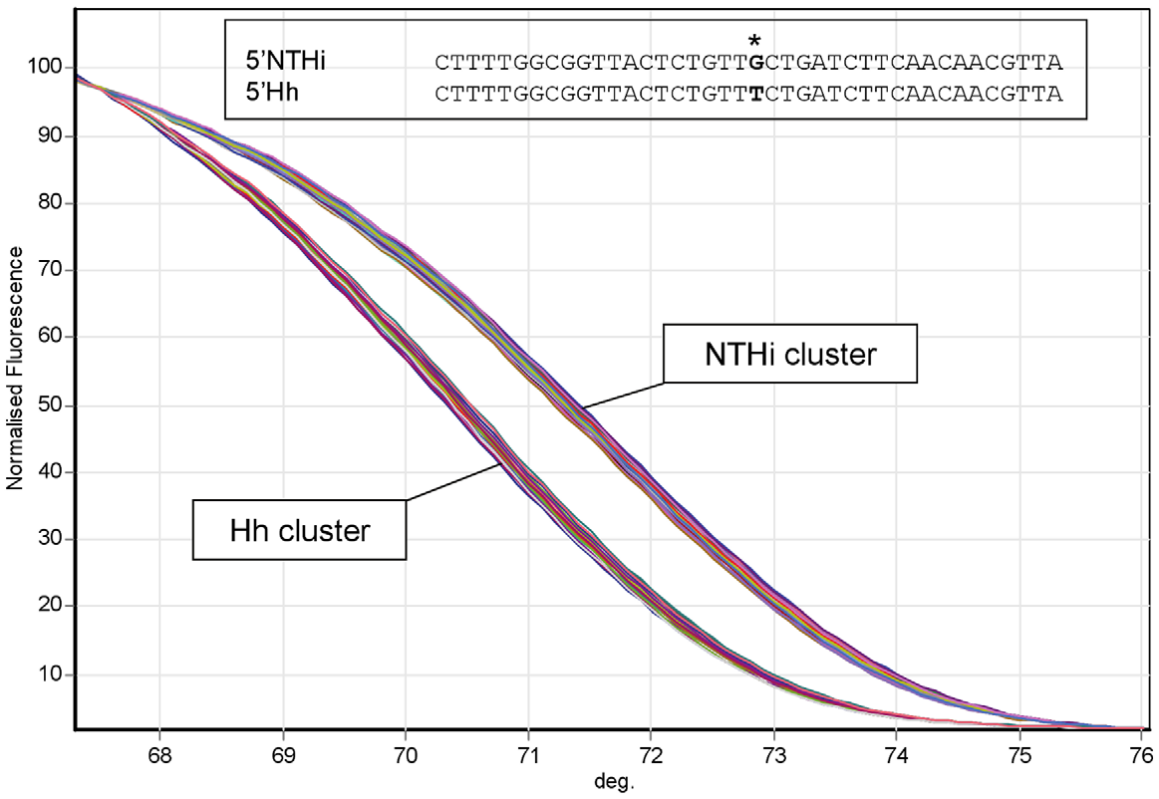
Differentiation of study isolates using *omp P6*-HRM. Analysis of publically available sequence identified a SNP in the *omp P6* genes of NTHi (G) and Hh (T) corresponding to nucleotide position 402465 of *H. influenzae* Rd KW20, Accession No. L42023. HRM of the 40 base pair amplicon (402446–402485) surrounding the SNP revealed 2 discrete melt profiles; one that clustered with reference *H. influenzae* (ATCC 19418) and another that clustered with reference Hh (ATCC 33390).

**Figure 3:**
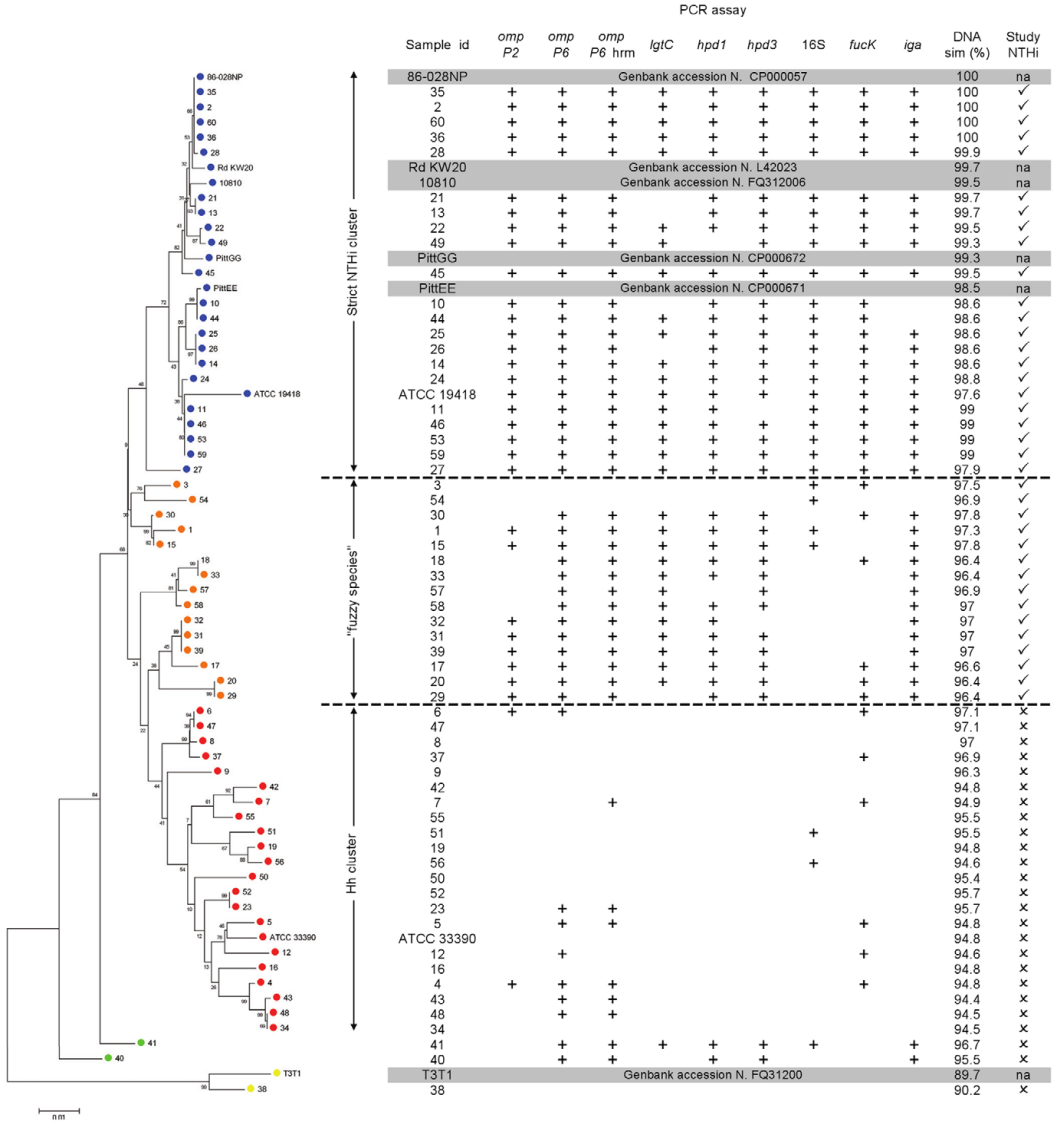
Neighbour-Joining dendogram and PCR results. The Neighbour-Joining dendogram of the concatenated 16S rRNA and *recA* gene sequences is displayed in conjunction with the PCR results and the percent DNA similarity (compared to *H. influenzae* 86-028NP). The tree is rooted by *H. parainfluenzae* as indicated by the yellow dots. The blue dots represent the strict NTHi group, the orange dots represent closely related phylogenetic variants, the red dots represent likely Hh isolates and the green dots represent variants related to *H. parainfluenzae*. Study-defined NTHi was based collectively on the phylogeny, PCR data and DNA similarity as is indicated on the right (✓ = NTHi or ✗ = Hh).

The 60 phenotypic NTHi study isolates were subsequently screened with 6 conventional PCR assays targeting the *omp P2*, *omp P6*, *lgtC*, 16S rRNA, *fucK* and *iga* genes, 2 real time PCR assays targeting the *hpd* gene, and a single PCR high resolution melt (PCR-HRM) assay designed to exploit a single nucleotide polymorphism (SNP) in the *omp P6* gene (*omp P6*-HRM) as shown in Figure 2. Overall the PCR assays revealed positivity ranging from 47% (16S rRNA) to 72% (*omp P6*). The *omp P6*-HRM separated the isolates into two distinct melt curves (Figure 2). The upper group of curves included the *H. influenzae* reference strain ATCC 19418 and the lower group of curves included the Hh reference strain ATCC 33390. The inclusion of standards in the real time PCR assays allowed an estimation of the limit of detection (LOD). For the *hpd#3* assay the LOD was below 10 copies per reaction at approximately cycle 36. The PCR results are displayed in conjunction with the 16S rRNA and *recA* concatenated phylogeny in Figure 3.

### Strict definition of NTHi

When assessed by the strict phylogenetic definition of NTHi shown in Figure 3 (Strict NTHi cluster), most PCR assays returned positive results. Only *lgtC* (4 negatives), *iga* (2 negatives), *hpd#1* and *hpd#3* (1 negative each) had less than 100% sensitivity. Against this strict definition, the 16S rRNA gene PCR assay demonstrated the greatest combination of sensitivity and specificity with a receiver operator characteristic (ROC) curve area of 91%, whereas all other assays had poor specificity (44%–70%). The inability of the 16S rRNA gene and *recA* sequence data to separate isolates into distinct species, and the detection of multiple NTHi target genes outside the strict NTHi group, led to the designation of several isolates as “fuzzy species” (Figure 3).

### Study-defined NTHi

For pragmatic assessment of the PCR assays, we enforced a study definition of NTHi. Isolates were interpreted as NTHi if they demonstrated a distinct phylogenetic grouping (16S rRNA and *recA* concatenated sequences), had approximately 97% DNA similarity or greater with the strict NTHi isolate 86-028NP (16S rRNA and *recA* concatenated sequences) and possessed most of the target genes (PCR results). Figure 3 displays our study-defined NTHi isolates which includes both the strict NTHi cluster and the fuzzy species.

### Assessment of PCR assays against study-defined NTHi

Sensitivity, specificity and ROC curve areas were calculated for each assay against study-defined NTHi. The *hpd#1*, *hpd#3* and *iga* PCR assays were equally most accurate, each returning a sensitivity, specificity and ROC curve area of 89%, 92% and 90% respectively (Table 2). The *omp P2* and *lgtC* assays had ROC curve areas of 86% and 88% respectively. The remainder of assays had ROC curve areas below 81%.

**Table 2:** Sensitivity, specificity and ROC curve areas of PCR assays for study-defined NTHi.

n = 60	*omp P2*	*omp P6*	*omp P6* HRM	*lgtC*	*hpd#1*	*hpd#3*	16S	*fucK*	*iga*
Sensitivity	80.6	94.4	94.4	80.6	88.9	88.9	69.4	75	88.9
Specificity	91.7	62.5	66.7	94.8	91.7	91.7	87.5	75	91.7
ROC area	0.86	0.78	0.81	0.88	0.90	0.90	0.78	0.75	0.90
[95% CI]	[0.77; 0.94]	[0.68; 0.89]	[0.70; 0.91]	[0.80; 0.96]	[0.83; 0.98]	[0.83; 0.98]	[0.68; 0.89]	[0.63; 0.86]	[0.83; 0.98]

### Reference isolates

Screening of the reference isolates showed that the *omp P2*, *omp P6*, *omp P6*-HRM, *lgtC*, *hpd#1* and *hpd#3* PCR-based assays were exclusively positive for the 2 reference *H. influenzae* strains (Table 3). The 16S rRNA, *fucK* and *iga* PCR assays were less specific, returning positive results for other organisms in the panel including *Haemophilus* species, *Pasteurella multocida*, *Neisseria meningitidis* and *Pseudomonas aeruginosa*. Interestingly, the *iga* PCR assay was positive for reference *H. parainfluenzae* (ATCC 7901) but not for study isolate 38, revealed to be *H. parainfluenzae* upon repeat microbiological examination.

**Table 3:** PCR screening of respiratory reference isolates.

Reference strain (ATCC)	*omp P2*	*omp P6*	*omp P6* HRM	*lgtC*	*hpd#1*	*hpd#3*	16S	*fucK*	*iga*
*Haemophilus influenzae* (19418 & 49274)	+	+	+	+	+	+	+	+	+
*Haemophilus haemolyticus* (33390)									
*Haemophilus parahaemolyticus* (10014)								+	
*Haemophilus parainfluenzae* (7901)								+	+
*Haemophilus aphrophilus* (19415)							+		
*Pasteurella multocida* (12945)							+	+	
*Neisseria meningitidis* (13090)									+
*Pseudomonas aeruginosa* (9027)							+		
*Streptococcus pneumoniae* (49619)									
*Moraxella catarrhalis* (8176)									
*Streptococcus pyogenes* (Local*)									
*Klebsiella pneumoniae* (Local*)									
*Staphylococcus aureus* (Local*)									

* Sourced from isolate collection at Menzies School of Health Research, Darwin, NT, Australia.

## Discussion

### Overview and significance

Accurate identification of NTHi is important to establish the relationship of this pathogen with carriage and infection. In the Northern Territory of Australia, where NTHi is a major cause of respiratory disease and otitis media, several randomised controlled trials are underway to assess the effect of antibiotics such as azithromycin, and vaccines including the pneumococcal *H. influenzae* protein D conjugate vaccine (PHiD-10CV; Synflorix®), on NTHi carriage. These studies rely on conventional microbiology. Failure to discriminate NTHi from its non-pathogenic relatives can result in a mismatch of cause (NTHi) and effect (respiratory disease and otitis media). We recommend the *hpd#3* assay for confirmation and future assessment of NTHi in carriage and disease.

### Sequence phylogeny

To challenge the PCR assays we selected phenotypic NTHi isolates with significant 16S rRNA gene variability as indicated by Murphy's 16S rRNA PCR [11] and this diversity was confirmed by the 16S rRNA and *recA* sequencing. The Neighbour-Joining radial phylogenetic trees in Figure 1 demonstrate an evolutionary continuum between NTHi and Hh that was unchanged using the alternative algorithms, Minimum Evolution or Maximum Parsimony. This “fuzziness” between species suggests that it may be impossible to differentiate NTHi from Hh without broader genetic interrogation. Other studies have used multilocus sequence analysis for improved *Haemophilus* species delineation [7], [8].

Considering NTHi and Hh are close evolutionary relatives that continue to inhabit an overlapping niche, maintaining their potential for genetic recombination, the lack of genetic distinction between these species is not surprising. The only obvious feature of the phylogeny is the strict cluster of NTHi isolates which might indicate that NTHi has a successful genetic formula for causing infection that Hh does not. This is supported by the finding that isolates collected as a consequence of clinical examination or from sterile sites are predominantly NTHi [8], [11], [14]. In this clinical context current microbiological techniques are generally adequate, however there have been recent reports of invasive disease caused by Hh [21].

### Novel PCR assays

The two novel assays that were designed for this study targeted *omp P6* and *omp P2*. The *omp P6*-HRM was set up to speciate NTHi from Hh via a SNP in the *omp P6* gene. Unfortunately, like the *omp P6* PCR, the *omp P6*-HRM over-represented NTHi. The apparent bimodal nature of the chosen *omp P6* SNP was not consistent with the overall genetic diversity that defined NTHi and Hh in this study. PCR-HRM is a powerful and cost effective method of identifying amplicon heterogeneity and careful target selection can allow successful species resolution [22]; a combination of several PCR-HRM assays might provide enhanced discrimination. The *omp P2* PCR assay was more accurate for detecting our study-defined NTHi with 81% sensitivity and 92% specificity and was among the best assays tested. This is comparable with a previous study, where *omp P2* PCR demonstrated 85% sensitivity for NTHi [12].

### PCR results

Six of the 9 PCR assays gave comparable results; the *omp P2*, *lgtC*, *hpd#1*, *hpd#3*, *fucK* and *iga* assays were positive for 50%–57% of isolates. The *omp P6* and *omp P6*-HRM assays tended to over represent NTHi (positive for 70% and 72% respectively) while the 16S rRNA gene assay detected only 47% of isolates producing results more aligned to the strict NTHi phylogeny. Most of the assays were positive for isolates beyond the strict NTHi cluster suggesting these fuzzy isolates were genetically more similar to NTHi than Hh. Thus, the study definition of NTHi considered the PCR results in addition to the phylogeny and similarity of the concatenated sequences (Figure 3).

Assessment of each PCR assay against the study definition of NTHi demonstrated that the 3 PCR assays, *hpd#1*, *hpd#3* and *iga*, were equally sensitive and specific against the 60 study isolates; however the *iga* PCR assay lacked specificity against the panel of reference organisms (Table 3). It should be noted that significant diversity exists among isolates of *Haemophilus* species and the results from the reference panel, comprising only 2 strains of NTHi and single strains of the other *Haemophilus* species, should be interpreted with caution.

In the recent publication by Wang *et al.* [12], the *hpd#3* assay was demonstrated to be highly specific but did detect 1 of 2 *H. aphrophilus* isolates among a reference panel of 61 respiratory organisms representing 21 different species. The reported LOD was 70 copies per PCR reaction when limited to 35 cycles. In our study, the *hpd#3* PCR assay did not detect any of the 12 non-*H. influenzae* reference organisms tested, including 1 *H. aphrophilus* isolate, and we consistently achieved a LOD of 10 copies per PCR reaction. When applied to predominantly invasive NTHi isolates, Wang showed that the *hpd#3* assay had a sensitivity of 98% (100/102). For our genetically diverse selection of study-defined NTHi isolates, the *hpd#3* assay was positive for 89% (33/37).

### Conclusion

In summary, no single gene target tested was able to unequivocally differentiate NTHi and Hh. Comparative genomic studies are required to identify the genetic determinants that enable NTHi to successfully invade sterile sites and cause disease.

The *hpd#3* probe-based real time PCR assay was the best assay tested, having a superior combination of sensitivity, specificity and LOD for NTHi. Furthermore, this assay has applicability to both clinical isolates and clinical specimens and can be used to quantify bacterial density making it a valuable tool for more accurate monitoring of NTHi in nasopharyngeal carriage, otitis media and respiratory infection.

## Materials and Methods

### Study isolates

This study tested DNA extracted from stored isolates obtained during a hospital-based surveillance study conducted in Western Australia from 2007 to 2009 [23]. Nasopharyngeal swabs were collected from children 6–36 months of age who had been anaesthetized for either tympanostomy tube insertion or general surgery. NTHi was identified by colony morphology and dependence on X and V growth factors. Isolates were defined as non-typeable based on a lack of agglutination with typing sera (Bactus AB). DNA was extracted using the Wizard SV gDNA kit according to manufacturer's instructions (Promega). The 60 isolates included in this study were defined by Murphy's dual 16S rRNA gene PCR algorithm [11] as NTHi (n = 22), Hh (n = 27), or equivocal (n = 11). This dual assay generates equivocal results in approximately 10% of clinical and surveillance isolates [11], [14].

### Sequence phylogeny

Partial sequencing of the 16S rRNA and *recA* genes, both commonly used for bacterial classification [7], [8], [24], was performed on the 60 clinical isolates and 2 reference strains (*H. influenzae* - ATCC 19418, and *H. haemolyticus* - ATCC 33390) to assist speciation. A complete 16S rRNA gene amplicon of approximately 1500 bp and a partial *recA* amplicon of approximately 600 bp were generated using PCR primers and methodology described elsewhere [11], [24]. Each amplicon was sequenced in singlicate using the reverse primer from the PCR. All sequencing was carried out by Macrogen, Korea. Six reference sequences were downloaded from Genbank [25] (www.ncbi.nlm.nih.gov – December 2010) to facilitate species identification (Table 1). Sequencing traces were assessed, cropped and exported for further analysis using the Lasergene software (DNASTAR, USA). Sequence alignments (ClustalW) and phylogenetic analyses (Neighbour-Joining, Minimum Evolution and Maximum Parsimony) were conducted using *MEGA* version 4 [26].

### Established PCR assays

A literature search was conducted to identify gene target candidates from established PCR-based assays with a high degree of accuracy for identification of NTHi, including discrimination from Hh. Seven targets were chosen on the basis of their demonstrated sensitivity and specificity for NTHi, and the PCR assays were conducted based on the published methods (Table 4). DNA for standards was extracted from the reference strain ATCC 19418 and a dilution series ranging from 10 to 100000 genome copies per reaction was used to estimate the LOD of real time PCR assays.

**Table 4:** Study primers and probes.

NTHi Target Gene		Sequence (5′ - 3′)	Length (bp)	[Primer/Probe] (nM)	Amplicon (bp)	Ref
*Established Assays*						
*fucK*	F	ACCACTTTCGGCGTGGATGG	20	500	343	[24]
	R	AAGATTTCCCAGGTGCCAGA	20	500		
*iga*	F	GTTCCACCACCTGCGCCTGCTAC	23	500	813*	[27]
	R	GTTATATTGCCCCTCGTTATTCA	23	500		
*omp P6*	F	CGGTTTTGATAAATATGACATTACT	25	300	182	[28]
	R	CTAAATAACCTTTAACTGCATCT	23	300		
*lgtC*	F	CGGACTGTCAGTCAGACAATG	21	500	839	[7]
	R	CTCAAAATGATCATACCAAGATG	23	500		
*hpd#1*	F	AGATTGGAAAGAAACACAAGAAAAAGA	27	300	113	[12]
	R	CACCATCGGCATATTTAACCACT	23	100		
	P	AAACATCCAATCG“T”AATTATAGTTTACCCAATAACCC **	37	200		
*hpd#3*	F	GGTTAAATATGCCGATGGTGTTG	23	100	151	[12]
	R	TGCATCTTTACGCACGGTGTA	21	300		
	P	TTGTGTACACTCCGT “T” GGTAAAAGAACTTGCAC **	33	100		
16S	F	TGACATCCTAAGAAGAGC	18	500	513	[11]
	R	GCAGGTTCCCTACGGTTA	18	500		
*Novel Assays*						
*omp P2*	F	GTTCACGTTTCCACATTAAAGC	22	500	186	This study
	R	CACGACCAAGTTTTACTTCAC	21	500		
*omp P6*(HRM)	F	CTTTTGGCGGTTACTCTGTT	20	500	40	This study
	R	TAACGTTGTTGAAGATCAG	19	500		

All product sizes are based on the NTHi Rd KW20 genome.

* product size varies considerably across strains.

** *hpd* probes were labelled with Hex at 5′-end, SpC3 at 3′-end, and Black Hole Quencher (BHQ) at the internal “T”. F (forward primer); R (reverse primer); P (probe); HRM (high resolution melt).

### Novel PCR assays

Publically available sequence data [25] (www.ncbi.nlm.nih.gov – September 2010) was utilised to design novel assays targeting the two outer membrane protein genes, *omp P2* and *omp P6*.

### *omp P2 PCR*

Primers (Table 4) were selected from the conserved regions of *omp P2* to generate a 186 base pair amplicon corresponding to nucleotide region 154223–154408 of the *H. influenzae* Rd KW20 complete genome, accession number L42023. PCR was performed using standard *Taq* PCR Core Kit reagents (Qiagen) with 0.5 µM of each primer and 1 µl of DNA extraction template in each 25 µl reaction. The annealing temperature was 55°C and cycling was repeated 35 times. PCR products were isolated by agarose gel electrophoresis, stained using SYBR® Safe DNA gel stain (Invitrogen), and visualised with the Gel Doc XR system in conjunction with Quantity One software (Bio-Rad).

### *omp P6-HRM*

The *omp P6* primers (Table 4) for PCR-HRM were selected around a conserved SNP (G for NTHi and T for Hh; as shown in Figure 2) corresponding to nucleotide 402465 (amplicon 402446–402485) of the *H. influenzae* Rd KW20 complete genome, accession number L42023. Real time PCR was performed using 5 µl of the 2× SensiMix™ SYBR® Green No-ROX reaction buffer (Quantace), 0.5 µM of each primer and 1 µl of DNA extraction template in each 10 µl reaction. Annealing was set at 58°C. Following 35 cycles of PCR, HRM was performed between 66°C and 76°C in 0.1°C increments for 2 seconds each. All thermocycling was done with the Rotor-Gene 6000 real time platform (Qiagen).

### Reference isolates

The PCR assays were also conducted on a panel of reference isolates of common respiratory bacterial species to determine their broader specificity. The following species were included: *H. influenzae*, *H. haemolyticus*, *H. parahaemolyticus*, *H. parainfluenzae*, *H. aphrophilus*, *P. multocida*, *N. meningitidis*, *P. aeruginosa*, *Streptococcus pneumoniae*, *Moraxella catarrhalis*, *Streptococcus pyogenes*, *Klebsiella pneumonia* and *Staphylococcus aureus*. Reference strains were sourced from either Microbiologics (Minnesota, USA) or locally from the culture collection at the Menzies School of Health Research (Northern Territory, Australia) as shown in Table 3.

### Statistical Analysis

Sequence phylogeny, PCR results, and DNA similarity were used to define NTHi for this study. The sensitivity and specificity of each PCR method for study NTHi was calculated and assessed by the area under the ROC curve. Analyses were conducted using STATA IC version 11 (StataCorp, Texas, USA).

## Supporting Information

Table S1 Partial 16S and recA sequence accession numbers.
(DOC)

## References

[1] Williams BG, Gouws E, Boschi-Pinto C, Bryce J, Dye C (2002). Estimates of world-wide distribution of child deaths from acute respiratory infections.. Lancet Infect Dis.

[2] Murphy TF (2003). Respiratory infections caused by non-typeable Haemophilus influenzae.. Curr Opin Infect Dis.

[3] Sethi S, Murphy TF (2001). Bacterial infection in chronic obstructive pulmonary disease in 2000: a state-of-the-art review.. Clin Microbiol Rev.

[4] Pichichero ME, Casey JR, Hoberman A, Schwartz R (2008). Pathogens causing recurrent and difficult-to-treat acute otitis media, 2003–2006.. Clin Pediatr (Phila).

[5] Kilian M (1976). A taxonomic study of the genus Haemophilus, with the proposal of a new species.. J Gen Microbiol.

[6] Corless CE, Guiver M, Borrow R, Edwards-Jones V, Fox AJ (2001). Simultaneous detection of Neisseria meningitidis, Haemophilus influenzae, and Streptococcus pneumoniae in suspected cases of meningitis and septicemia using real-time PCR.. J Clin Microbiol.

[7] McCrea KW, Xie J, LaCross N, Patel M, Mukundan D (2008). Relationships of nontypeable Haemophilus influenzae strains to hemolytic and nonhemolytic Haemophilus haemolyticus strains.. J Clin Microbiol.

[8] Norskov-Lauritsen N, Overballe MD, Kilian M (2009). Delineation of the species Haemophilus influenzae by phenotype, multilocus sequence phylogeny, and detection of marker genes.. J Bacteriol.

[9] Norskov-Lauritsen N (2009). Detection of cryptic genospecies misidentified as Haemophilus influenzae in routine clinical samples by assessment of marker genes fucK, hap, and sodC.. J Clin Microbiol.

[10] Prymula R, Kriz P, Kaliskova E, Pascal T, Poolman J (2009). Effect of vaccination with pneumococcal capsular polysaccharides conjugated to Haemophilus influenzae-derived protein D on nasopharyngeal carriage of Streptococcus pneumoniae and H. influenzae in children under 2 years of age.. Vaccine.

[11] Murphy TF, Brauer AL, Sethi S, Kilian M, Cai X (2007). Haemophilus haemolyticus: a human respiratory tract commensal to be distinguished from Haemophilus influenzae.. J Infect Dis.

[12] Wang X, Mair R, Hatcher C, Theodore MJ, Edmond K (2011). Detection of bacterial pathogens in Mongolia meningitis surveillance with a new real-time PCR assay to detect Haemophilus influenzae.. Int J Med Microbiol.

[13] Abdeldaim GM, Stralin K, Kirsebom LA, Olcen P, Blomberg J (2009). Detection of Haemophilus influenzae in respiratory secretions from pneumonia patients by quantitative real-time polymerase chain reaction.. Diagn Microbiol Infect Dis.

[14] Kirkham LA, Wiertsema SP, Mowe EN, Bowman JM, Riley TV (2010). Nasopharyngeal carriage of Haemophilus haemolyticus in otitis-prone and healthy children.. J Clin Microbiol.

[15] Morris PS, Leach AJ, Silberberg P, Mellon G, Wilson C (2005). Otitis media in young Aboriginal children from remote communities in Northern and Central Australia: a cross-sectional survey.. BMC Pediatr.

[16] O'Grady KA, Lee KJ, Carlin JB, Torzillo PJ, Chang AB (2010). Increased risk of hospitalization for acute lower respiratory tract infection among Australian indigenous infants 5–23 months of age following pneumococcal vaccination: a cohort study.. Clin Infect Dis.

[17] Binks MJ, Christensen P, Marsh R, Morris PS, Leach AJ (2011). Dominance of H. influenzae, but not S. pneumoniae or M. catarrhalis, in ear discharge compared to the nasopharynx in paired swabs..

[18] Hare KM, Grimwood K, Leach AJ, Smith-Vaughan H, Torzillo PJ (2010). Respiratory Bacterial Pathogens in the Nasopharynx and Lower Airways of Australian Indigenous Children with Bronchiectasis.. J Pediatr.

[19] Stubbs E, Hare K, Wilson C, Morris P, Leach AJ (2005). Streptococcus pneumoniae and noncapsular Haemophilus influenzae nasal carriage and hand contamination in children: a comparison of two populations at risk of otitis media.. Pediatr Infect Dis J.

[20] Leach A, Wood Y, Gadil E, Stubbs E, Morris P (2008). Topical ciprofloxin versus topical framycetin-gramicidin-dexamethasone in Australian aboriginal children with recently treated chronic suppurative otitis media: a randomized controlled trial.. Pediatr Infect Dis J.

[21] Jordan IK, Conley AB, Antonov IV, Arthur RA, Cook ED (2011). Genome sequences for five strains of the emerging pathogen Haemophilus haemolyticus.. J Bacteriol.

[22] Lilliebridge RA, Tong SYC, Giffard PM, Holt DC (2011). The utility of high-resolution melting analysis of SNP nucleated PCR amplicons—an MLST based Staphylococcus aureus typing scheme.. PLoS One.

[23] Wiertsema SP, Kirkham LA, Corscadden KJ, Mowe EN, Bowman JM (2011). Predominance of nontypeable Haemophilus influenzae in children with otitis media following introduction of a 3+0 pneumococcal conjugate vaccine schedule.. Vaccine.

[24] Meats E, Feil EJ, Stringer S, Cody AJ, Goldstein R (2003). Characterization of encapsulated and noncapsulated Haemophilus influenzae and determination of phylogenetic relationships by multilocus sequence typing.. J Clin Microbiol.

[25] Benson DA, Karsch-Mizrachi I, Lipman DJ, Ostell J, Wheeler DL (2008). GenBank.. Nucleic Acids Res.

[26] Tamura K, Dudley J, Nei M, Kumar S (2007). MEGA4: Molecular Evolutionary Genetics Analysis (MEGA) software version 4.0.. Mol Biol Evol.

[27] Vitovski S, Dunkin KT, Howard AJ, Sayers JR (2002). Nontypeable Haemophilus influenzae in carriage and disease: a difference in IgA1 protease activity levels.. JAMA.

[28] Vu HT, Yoshida LM, Suzuki M, Nguyen HA, Nguyen CD (2011). Association between nasopharyngeal load of Streptococcus pneumoniae, viral coinfection, and radiologically confirmed pneumonia in Vietnamese children.. Pediatr Infect Dis J.

