# Supplementary material for: Molecular Surveillance of True Nontypeable Haemophilus influenzae: An Evaluation of PCR Screening Assays
Source: PLoS One. 2012 Mar 28;7(3):e34083. doi: 10.1371/journal.pone.0034083 (PMC3314702; doi:10.1371/journal.pone.0034083)
Supplement: Table S1 — Partial 16S and recA sequence accession numbers. (DOC) [file pone.0034083.s001.doc]

**Table S1. Partial 16S and recA sequence accession numbers.**

| **Gene** | **Bacterial species** | **Strain** | **Accession#** | **Serotype** | **Isolation source** | **Country** |
| --- | --- | --- | --- | --- | --- | --- |
| ***16S*** | *H. influenzae* | UWA: 26 | JN990145 | Non typeable | Nasopharynx | Australia |
|  | *H. influenzae* | UWA: 16 | JN990146 | Non typeable | Nasopharynx | Australia |
|  | *H. influenzae* | UWA: 41 | JN990147 | Non typeable | Nasopharynx | Australia |
|  | *H. influenzae* | UWA: 18 | JN990148 | Non typeable | Nasopharynx | Australia |
|  | *H. influenzae* | UWA: 6 | JN990149 | Non typeable | Nasopharynx | Australia |
|  | *H. influenzae* | UWA: 44 | JN990150 | Non typeable | Nasopharynx | Australia |
|  | *H. influenzae* | UWA: 24 | JN990151 | Non typeable | Nasopharynx | Australia |
|  | *H. influenzae* | UWA: 14 | JN990152 | Non typeable | Nasopharynx | Australia |
|  | *H. influenzae* | UWA: 42 | JN990153 | Non typeable | Nasopharynx | Australia |
|  | *H. influenzae* | UWA: 40 | JN990154 | Non typeable | Nasopharynx | Australia |
|  | *H. influenzae* | UWA: 19 | JN990155 | Non typeable | Nasopharynx | Australia |
|  | *H. influenzae* | UWA: 8 | JN990156 | Non typeable | Nasopharynx | Australia |
|  | *H. influenzae* | UWA: 39 | JN990157 | Non typeable | Nasopharynx | Australia |
|  | *H. influenzae* | UWA: 53 | JN990158 | Non typeable | Nasopharynx | Australia |
|  | *H. influenzae* | UWA: 32 | JN990159 | Non typeable | Nasopharynx | Australia |
|  | *H. influenzae* | UWA: 21 | JN990160 | Non typeable | Nasopharynx | Australia |
|  | *H. influenzae* | UWA: 30 | JN990161 | Non typeable | Nasopharynx | Australia |
|  | *H. influenzae* | UWA: 56 | JN990162 | Non typeable | Nasopharynx | Australia |
|  | *H. influenzae* | UWA: 36 | JN990163 | Non typeable | Nasopharynx | Australia |
|  | *H. influenzae* | UWA: 16 | JN990164 | Non typeable | Nasopharynx | Australia |
|  | *H. influenzae* | UWA: 38 | JN990165 | Non typeable | Nasopharynx | Australia |
|  | *H. influenzae* | UWA: 54 | JN990166 | Non typeable | Nasopharynx | Australia |
|  | *H. influenzae* | UWA: 25 | JN990167 | Non typeable | Nasopharynx | Australia |
|  | *H. influenzae* | UWA: 22 | JN990168 | Non typeable | Nasopharynx | Australia |
|  | *H. influenzae* | UWA: 33 | JN990169 | Non typeable | Nasopharynx | Australia |
|  | *H. influenzae* | UWA: 55 | JN990170 | Non typeable | Nasopharynx | Australia |
|  | *H. influenzae* | UWA: 47 | JN990171 | Non typeable | Nasopharynx | Australia |
|  | *H. influenzae* | UWA: 17 | JN990172 | Non typeable | Nasopharynx | Australia |
|  | *H. influenzae* | UWA: 37 | JN990173 | Non typeable | Nasopharynx | Australia |
|  | *H. influenzae* | UWA: 27 | JN990174 | Non typeable | Nasopharynx | Australia |
|  | *H. influenzae* | UWA: 13 | JN990175 | Non typeable | Nasopharynx | Australia |
|  | *H. influenzae* | UWA: 48 | JN990176 | Non typeable | Nasopharynx | Australia |
|  | *H. influenzae* | UWA: 5 | JN990177 | Non typeable | Nasopharynx | Australia |
|  | *H. influenzae* | UWA: 20 | JN990178 | Non typeable | Nasopharynx | Australia |
|  | *H. influenzae* | UWA: 52 | JN990179 | Non typeable | Nasopharynx | Australia |
|  | *H. influenzae* | UWA: 1 | JN990180 | Non typeable | Nasopharynx | Australia |
|  | *H. influenzae* | UWA: 2 | JN990181 | Non typeable | Nasopharynx | Australia |
|  | *H. influenzae* | UWA: 43 | JN990182 | Non typeable | Nasopharynx | Australia |
|  | *H. influenzae* | UWA: 51 | JN990183 | Non typeable | Nasopharynx | Australia |
|  | *H. influenzae* | UWA: 4 | JN990184 | Non typeable | Nasopharynx | Australia |
|  | *H. influenzae* | UWA: 12 | JN990185 | Non typeable | Nasopharynx | Australia |
|  | *H. influenzae* | UWA: 10 | JN990186 | Non typeable | Nasopharynx | Australia |
|  | *H. influenzae* | UWA: 9 | JN990187 | Non typeable | Nasopharynx | Australia |
|  | *H. influenzae* | UWA: 23 | JN990188 | Non typeable | Nasopharynx | Australia |
|  | *H. influenzae* | UWA: 59 | JN990189 | Non typeable | Nasopharynx | Australia |
|  | *H. influenzae* | UWA: 34 | JN990190 | Non typeable | Nasopharynx | Australia |
|  | *H. influenzae* | UWA: 28 | JN990191 | Non typeable | Nasopharynx | Australia |
|  | *H. influenzae* | UWA: 60 | JN990192 | Non typeable | Nasopharynx | Australia |
|  | *H. influenzae* | UWA: 31 | JN990193 | Non typeable | Nasopharynx | Australia |
|  | *H. influenzae* | UWA: 46 | JN990194 | Non typeable | Nasopharynx | Australia |
|  | *H. influenzae* | UWA: 11 | JN990195 | Non typeable | Nasopharynx | Australia |
|  | *H. influenzae* | UWA: 35 | JN990196 | Non typeable | Nasopharynx | Australia |
|  | *H. influenzae* | UWA: 50 | JN990197 | Non typeable | Nasopharynx | Australia |
|  | *H. influenzae* | UWA: 7 | JN990198 | Non typeable | Nasopharynx | Australia |
|  | *H. influenzae* | UWA: 15 | JN990199 | Non typeable | Nasopharynx | Australia |
|  | *H. influenzae* | UWA: 29 | JN990200 | Non typeable | Nasopharynx | Australia |
|  | *H. influenzae* | UWA: 49 | JN990201 | Non typeable | Nasopharynx | Australia |
|  | *H. influenzae* | UWA: 58 | JN990202 | Non typeable | Nasopharynx | Australia |
|  | *H. influenzae* | UWA: 57 | JN990203 | Non typeable | Nasopharynx | Australia |
|  | *H. influenzae* | UWA: 45 | JN990204 | Non typeable | Nasopharynx | Australia |
| **Gene** | **Bacterial species** | **Strain** | **Accession#** | **Serotype** | **Isolation source** | **Country** |
| ***recA*** | *H. influenzae* | UWA: 26 | JN990205 | Non typeable | Nasopharynx | Australia |
|  | *H. influenzae* | UWA: 43 | JN990206 | Non typeable | Nasopharynx | Australia |
|  | *H. influenzae* | UWA: 5 | JN990207 | Non typeable | Nasopharynx | Australia |
|  | *H. influenzae* | UWA: 31 | JN990208 | Non typeable | Nasopharynx | Australia |
|  | *H. influenzae* | UWA: 48 | JN990209 | Non typeable | Nasopharynx | Australia |
|  | *H. influenzae* | UWA: 15 | JN990210 | Non typeable | Nasopharynx | Australia |
|  | *H. influenzae* | UWA: 32 | JN990211 | Non typeable | Nasopharynx | Australia |
|  | *H. influenzae* | UWA: 47 | JN990212 | Non typeable | Nasopharynx | Australia |
|  | *H. influenzae* | UWA: 54 | JN990213 | Non typeable | Nasopharynx | Australia |
|  | *H. influenzae* | UWA: 25 | JN990214 | Non typeable | Nasopharynx | Australia |
|  | *H. influenzae* | UWA: 40 | JN990215 | Non typeable | Nasopharynx | Australia |
|  | *H. influenzae* | UWA: 51 | JN990216 | Non typeable | Nasopharynx | Australia |
|  | *H. influenzae* | UWA: 39 | JN990217 | Non typeable | Nasopharynx | Australia |
|  | *H. influenzae* | UWA: 23 | JN990218 | Non typeable | Nasopharynx | Australia |
|  | *H. influenzae* | UWA: 14 | JN990219 | Non typeable | Nasopharynx | Australia |
|  | *H. influenzae* | UWA: 45 | JN990220 | Non typeable | Nasopharynx | Australia |
|  | *H. influenzae* | UWA: 44 | JN990221 | Non typeable | Nasopharynx | Australia |
|  | *H. influenzae* | UWA: 57 | JN990222 | Non typeable | Nasopharynx | Australia |
|  | *H. influenzae* | UWA: 16 | JN990223 | Non typeable | Nasopharynx | Australia |
|  | *H. influenzae* | UWA: 38 | JN990224 | Non typeable | Nasopharynx | Australia |
|  | *H. influenzae* | UWA: 60 | JN990225 | Non typeable | Nasopharynx | Australia |
|  | *H. influenzae* | UWA: 17 | JN990226 | Non typeable | Nasopharynx | Australia |
|  | *H. influenzae* | UWA: 5 | JN990227 | Non typeable | Nasopharynx | Australia |
|  | *H. influenzae* | UWA: 21 | JN990228 | Non typeable | Nasopharynx | Australia |
|  | *H. influenzae* | UWA: 18 | JN990229 | Non typeable | Nasopharynx | Australia |
|  | *H. influenzae* | UWA: 2 | JN990230 | Non typeable | Nasopharynx | Australia |
|  | *H. influenzae* | UWA: 46 | JN990231 | Non typeable | Nasopharynx | Australia |
|  | *H. influenzae* | UWA: 22 | JN990232 | Non typeable | Nasopharynx | Australia |
|  | *H. influenzae* | UWA: 9 | JN990233 | Non typeable | Nasopharynx | Australia |
|  | *H. influenzae* | UWA: 52 | JN990234 | Non typeable | Nasopharynx | Australia |
|  | *H. influenzae* | UWA: 42 | JN990235 | Non typeable | Nasopharynx | Australia |
|  | *H. influenzae* | UWA: 12 | JN990236 | Non typeable | Nasopharynx | Australia |
|  | *H. influenzae* | UWA: 28 | JN990237 | Non typeable | Nasopharynx | Australia |
|  | *H. influenzae* | UWA: 13 | JN990238 | Non typeable | Nasopharynx | Australia |
|  | *H. influenzae* | UWA: 36 | JN990239 | Non typeable | Nasopharynx | Australia |
|  | *H. influenzae* | UWA: 24 | JN990240 | Non typeable | Nasopharynx | Australia |
|  | *H. influenzae* | UWA: 59 | JN990241 | Non typeable | Nasopharynx | Australia |
|  | *H. influenzae* | UWA: 37 | JN990242 | Non typeable | Nasopharynx | Australia |
|  | *H. influenzae* | UWA: 33 | JN990243 | Non typeable | Nasopharynx | Australia |
|  | *H. influenzae* | UWA: 20 | JN990244 | Non typeable | Nasopharynx | Australia |
|  | *H. influenzae* | UWA: 34 | JN990245 | Non typeable | Nasopharynx | Australia |
|  | *H. influenzae* | UWA: 19 | JN990246 | Non typeable | Nasopharynx | Australia |
|  | *H. influenzae* | UWA: 30 | JN990247 | Non typeable | Nasopharynx | Australia |
|  | *H. influenzae* | UWA: 6 | JN990248 | Non typeable | Nasopharynx | Australia |
|  | *H. influenzae* | UWA: 41 | JN990249 | Non typeable | Nasopharynx | Australia |
|  | *H. influenzae* | UWA: 55 | JN990250 | Non typeable | Nasopharynx | Australia |
|  | *H. influenzae* | UWA: 29 | JN990251 | Non typeable | Nasopharynx | Australia |
|  | *H. influenzae* | UWA: 4 | JN990252 | Non typeable | Nasopharynx | Australia |
|  | *H. influenzae* | UWA: 50 | JN990253 | Non typeable | Nasopharynx | Australia |
|  | *H. influenzae* | UWA: 49 | JN990254 | Non typeable | Nasopharynx | Australia |
|  | *H. influenzae* | UWA: 8 | JN990255 | Non typeable | Nasopharynx | Australia |
|  | *H. influenzae* | UWA: 27 | JN990256 | Non typeable | Nasopharynx | Australia |
|  | *H. influenzae* | UWA: 53 | JN990257 | Non typeable | Nasopharynx | Australia |
|  | *H. influenzae* | UWA: 11 | JN990258 | Non typeable | Nasopharynx | Australia |
|  | *H. influenzae* | UWA: 10 | JN990259 | Non typeable | Nasopharynx | Australia |
|  | *H. influenzae* | UWA: 3 | JN990260 | Non typeable | Nasopharynx | Australia |
|  | *H. influenzae* | UWA: 7 | JN990261 | Non typeable | Nasopharynx | Australia |
|  | *H. influenzae* | UWA: 35 | JN990262 | Non typeable | Nasopharynx | Australia |
|  | *H. influenzae* | UWA: 58 | JN990263 | Non typeable | Nasopharynx | Australia |
|  | *H. influenzae* | UWA: 1 | JN990264 | Non typeable | Nasopharynx | Australia |
